# Aberrant role of ALK in tau proteinopathy through autophagosomal dysregulation

**DOI:** 10.1038/s41380-020-01003-y

**Published:** 2021-01-15

**Authors:** Jisu Park, Hyunwoo Choi, Young Doo Kim, Seo-Hyun Kim, Youbin Kim, Youngdae Gwon, Dong Young Lee, Sung-Hye Park, Won Do Heo, Yong-Keun Jung

**Affiliations:** 1 School of Biological Science, Seoul National University, 1 Gwanak-ro, Gwanak-gu, Seoul, 08826 Republic of Korea; 2 Department of Neuropsychiatry, Seoul National University College of Medicine, Seoul, 13080 Republic of Korea; 3 Department of Pathology, Seoul National University College of Medicine, Seoul, 03080 Republic of Korea; 4 Department of Biological Sciences, Korea Advanced Institute of Science and Technology, Kuseong-dong, Yuseong-ku, Daejeon, 305-701 Korea

**Keywords:** Neuroscience, Cell biology

## Abstract

Proteinopathy in neurodegenerative diseases is typically characterized by deteriorating activity of specific protein aggregates. In tauopathies, including Alzheimer’s disease (AD), tau protein abnormally accumulates and induces dysfunction of the affected neurons. Despite active identification of tau modifications responsible for tau aggregation, a critical modulator inducing tau proteinopathy by affecting its protein degradation flux is not known. Here, we report that anaplastic lymphoma kinase (ALK), a receptor tyrosine kinase, is crucial for the tau-mediated AD pathology. ALK caused abnormal accumulation of highly phosphorylated tau in the somatodendritic region of neurons through its tyrosine kinase activity. ALK-induced LC3-positive axon swelling and loss of spine density, leading to tau-dependent neuronal degeneration. Notably, ALK activation in neurons impaired Stx17-dependent autophagosome maturation and this defect was reversed by a dominant-negative Grb2. In a *Drosophila melanogaster* model, transgenic flies neuronally expressing active *Drosophila* Alk exhibited the aggravated tau rough eye phenotype with retinal degeneration and shortened lifespan. In contrast, expression of kinase-dead Alk blocked these phenotypes. Consistent with the previous RNAseq analysis showing upregulation of ALK expression in AD [1], ALK levels were significantly elevated in the brains of AD patients showing autophagosomal defects. Injection of an ALK.Fc-lentivirus exacerbated memory impairment in 3xTg-AD mice. Conversely, pharmacologic inhibition of ALK activity with inhibitors reversed the memory impairment and tau accumulation in both 3xTg-AD and tauC3 (caspase-cleaved tau) transgenic mice. Together, we propose that aberrantly activated ALK is a bona fide mediator of tau proteinopathy that disrupts autophagosome maturation and causes tau accumulation and aggregation, leading to neuronal dysfunction in AD.

## Introduction

Accumulation of protein aggregates is a shared feature of neurodegenerative disorders. Among these are the tauopathies, which exhibit accumulation of tau protein [2] and include Alzheimer’s disease (AD), progressive supranuclear palsy, Pick’s disease, corticobasal degeneration, and frontotemporal dementia with parkinsonism linked to chromosome 17 (FTDP-17) [3–6]. AD, the most common cause of dementia, is characterized by the deposition of intracellular tau tangles. Abnormally phosphorylated tau is cleared via an autophagic process involving autophagosome-dependent, lysosome-mediated degradation. Otherwise, the protein mislocalizes and accumulates within neurons [7]. Although it has been proposed that tau functions exclusively downstream of amyloid-beta (Aβ) [8, 9], an increasing number of studies have found that tau is able to exert adverse effects on neurons independently of or dominantly over Aβ [10]. Moreover, ApoE and Triggering Receptor Expressed On Myeloid Cells 2 (TREM2), the most well-known risk factors for sporadic AD, are highly associated with tau-mediated pathology, deteriorating tau-induced neurotoxicity in a mouse model of tauopathy [11], and worsening gliosis and inflammation related to tau-mediated neuronal damage [12, 13]. Despite many studies focusing on tau modification and aggregation, a crucial receptor that regulates tau accumulation, clearance, and pathology beyond that mentioned above remains elusive.

Anaplastic lymphoma kinase (ALK) is a receptor tyrosine kinase belonging to the insulin receptor superfamily [14, 15]. ALK has a restricted distribution in mammals, being found at significant levels mainly in the central nervous system during development [14, 16]. A low level of ALK expression is maintained in the adult brain and *Alk* knockout mice display a full lifespan [17]. Aberrant ALK activity has been strongly implicated in the oncogenesis of human cancer as a fusion protein in inflammatory myofibroblastic tumors, diffuse large B-cell lymphoma and anaplastic large-cell lymphoma, or through mutations in the full-length protein in hereditary familial neuroblastoma [18–22]. This makes ALK a therapeutic target in cancer [23–25]. More recent evidence also indicates that ALK may regulate the STING pathway and innate immune responses [26]. However, its role in neurodegeneration and AD pathology is not known.

To screen tau aggregation regulator, we developed a cell-based tau aggregation assay using tauC3, a caspase-cleaved form of human 0N4R tau (1–420), which is found in the brains of AD patients [27] and aggregates faster than wild-type tau in vitro [28]. Here, we report that ALK mediates tau pathology. ALK activation induced tau phosphorylation and impaired autophagosome maturation, thereby preventing degradation and accelerating aggregation of abnormally phosphorylated tau. In *Drosophila melanogaster*, ALK exacerbated the tau rough eye phenotype. In the 3xTg-AD and tauC3 mouse AD models, ALK exacerbated tau-related memory deficits, while its pharmacological inhibition restored memory in the model mice. Our results define a crucial role of ALK in tau-mediated neurodegeneration and provide insight into the pathogenesis of AD and a new approach to AD therapeutics.

## Materials and methods

### Cell culture

HT22 and SH-SY5Y cells were cultured in Dulbecco’s Modified Eagles Medium (Invitrogen) with 10% (v/v) fetal bovine serum (Gibco). Stably tau-expressing HT22 cells were maintained in media supplemented with 200 μg/ml hygromycin B (Clontech) and tau expression was induced by treatment with 1 μg/ml doxycycline (Sigma). Primary mouse hippocampal and cortical neurons were cultured at embryonic day 17. Briefly, mouse brain hippocampi and cortices were trypsinized and neuronal cells were transferred to neurobasal medium containing B27 serum supplement (Invitrogen) and seeded on 12-well tissue culture plates at a density of 2 × 10^6^ cells per well.

### Generation of tau stable cell line

HT22 cells were transfected with pBIG2i-tau for 36 h and then cultivated in a selection medium containing 200 μg/ml hygromycin B for 2 weeks. A single cell was further cultivated to form stable cell clones, and the expression level of each was analyzed by western blotting in the presence of doxycycline. SH-SY5Y cells were transfected with GFP-tau and cultivated in media supplemented with 1 mg/ml G418 for 2 weeks to generate a mixed cell population. A single cell was grown to form a stable cell line.

### Construction of plasmids

Tau (2N4R), the human longest form, in mammalian expression pCI vector was provided by Dr. Akihiko Takashima (RIKEN, Japan). Tau cDNA was subcloned into pcDNA3-HA (Invitrogen), pGFP (Clontech), and pBIG2i containing the tetracycline-regulated promoter for regulating the expression of an inserted gene and the selection marker for hygromycin B to generate HA or GFP fusion protein (pHA-tau, pGFP-tau, and pBIG2i-tau, respectively) [29]. Mouse ALK (mALK; NCBI Reference Sequence, NM_007439.2) in mammalian expression pME18S-FL3 (pmALK) was obtained from Dr. Tadashi Yamamoto (University of Tokyo, Japan) [14]. Full-length human ALK (ALK; NCBI Reference Sequence, NM_001353765.2) and kinase-dead mutant (ALK KD; originally named ALKΔATP) in which the invariant lysine residue (K1150) located in the ATP-binding region was mutated to alanine were provided by Dr. Anton Wellstein (Georgetown University, DC) [30] and subcloned into pcDNA3.1/Myc-His (pALK and pALK KD, respectively). ALK chimera (ALK.Fc) containing mouse IgG 2b Fc domain instead of the extracellular domain of the receptor in pcDNA3.1 and its kinase-defective form (ALK.Fc KD; originally named ALK*.Fc) in pcDNA3.1 (pALK.Fc and pALK.Fc KD, respectively) were gifts from Dr. Marc Vigny (INSERM U440, Paris) [31]. ALK.Fc and ALK.Fc KD were subcloned into lentiviral pCSII-EF-MCS-IRES2-Venus vector for the production of lentivirus (pLenti-ALK.Fc and pLenti-ALK.Fc KD, respectively).

### Transfection, cell death assessment, and viability assay

Expression plasmids were transfected using Lipofectamine 2000 (Invitrogen) or Lipofector-pMAX (Aptabio) following the manufacturer’s instructions. Cells were stained with 2 μg/ml propidium iodid (PI; Sigma) for 5 min or 3 μg/ml ethidium homodimer (EtHD; Molecular Probes) for 10 min. Cell death was assessed by counting cells with condensed, or fragmented nuclei or EtHD18-positive cells.

### Human brain samples and ethical statement

Anterior hippocampal tissues were a kind gift from the Harvard Brain Tissue Resource Center (McLean Hospital). Hippocampal tissues of patients with AD and controls were lyzed in ice-cold Tris-buffered saline (TBS) buffer [20 mM Tris-HCl (pH 7.4), 150 mM NaCl and protease inhibitor cocktails] and then the lysates were centrifuged at 13,000 rpm for 20 min at 4 °C. The supernatants were subjected to SDS-PAGE. This study was approved by the Institutional Review Board of Seoul National University.

**Table:** 

Diagnosis	Age	Sex	PMI^a^
Normal	89	F	14.12
Normal	82	F	15.7
Normal	81	F	26.18
Normal	76	M	12.25
Normal	73	M	24
Brrak V	92	F	5
Brrak V	83	F	19.25
Brrak V	77	M	11.05
Brrak V	95	F	7.58
Brrak V	89	F	27.00
Brrak VI	97	F	12.08
Brrak VI	83	M	25.41
Brrak VI	82	F	19.17
Brrak VI	68	M	8.62
Brrak VI	59	F	22.42

^a^*PMI* Post-mortem interval (h).

### Preparation of tissue lysates from mouse brain and fly heads

Tissue regions of brain were homogenized in TBS [20 mM Tris-Cl (pH 7.4), 150 mM NaCl, 1% Triton X-100, 1 mM Na_3_VO_4_, 1 mM NaF, 1 mM PMSF and 1 μg/ml each of aprotinin, leupeptin and pepstatin A]. Fly heads were homogenized in homogenization buffer [50 mM Tris-Cl (pH 8.0), 150 mM NaCl, 1% Triton X-100, 10% sucrose, 1 mM Na_3_VO_4_, 1 mM NaF, 1 mM PMSF and 1 μg/ml each of aprotinin, leupeptin and pepstatin A]. The homogenates were centrifuged at 15,000 × *g* for 30 min and protein concentrations in resultant supernatants were determined using Bradford assay (Bio-Rad).

### Western blot analysis

Cells were lysed in ice-cold RIPA buffer [50 mM Tris-Cl (pH 8.0), 15 mM NaCl, 1% Triton X-100, 0.5% sodium deoxycholate, 0.1% SDS, 1 mM PMSF and 1 μg/ml each of aprotinin, leupeptin and pepstatin A]. Cell lysates were clarified by centrifugation at 13,000 × *g* for 10 min, diluted in 2× SDS loading buffer [100 mM Tris-Cl (pH 6.8), 4% SDS, 20% glycerol, 0.01% bromophenol blue and 10% β-mercaptoethanol], resolved by SDS-PAGE and transferred onto PVDF.

### Immunocytochemistry

Cells were fixed with 4% paraformaldehyde for 20 min and then permeabilized with 0.1% Triton X-100 for 5 min, followed by blocking with 4% BSA. Images were obtained by using a confocal laser scanning microscope (Carl Zeiss, LSM700).

### Immunohistochemistry of human brain tissue

Human brain tissues were provided by the Brain Bank of Seoul National University Hospital Biomedical Research Institute. Hippocampal sections from AD patients were retrieved with 10% formic acid for 15 min at 37 °C and then blocked with 5% BSA. Blocked sections were stained with antibodies against ALK and p-Tau S386. Antibodies for ALK (DAKO) and p-Tau S386 (Invitrogen) were used to detect human ALK and neurofibrillary tangles, respectively.

### Antibodies

The following anti-tau antibodies were used: HT7 (human-specific Tau; Invitrogen, #MN-1000), Tau-5 (total tau; Biosource, #AHB0042), Tau-12 (total tau; generously provided by Dr. Lester Binder, Northwestern University, IL), TG5 and DA9 (total tau; generously provided by Dr. Peter Davies, Albert Einstein College of Medicine, NY), p-Tau Ser396 (Invitrogen, #44-752G), PHF-1 (p-Ser396/404; Davies), CP13 (p-S202; Davies), AT100 (p-Ser212/214; Pierce, #P10636), AT180 (pThr231/Ser235; Innogenetics, #90337), 12E8 (p-Ser262/356; generously provided by Dr. Peter Seubert, Elan Pharmaceuticals), Tau-1 (dephosphorylated Ser195/198/199/202; Chemicon, #MAB3420), MC-1 (conformation dependent antibody; Davies), LC3 (Novus Biologicals, #NB600–1384), SQSTM1 (Abnova, #H00008878-M01), pALK (p-Tyr1604; Cell signaling, #3341), ALK1 (BD Pharmingen, #559254), ALK (Invitrogen, #51–3900), ALK (DAKO, #IS64130–2), α-tubulin (Sigma, #T5168), β-actin (Sigma, #A2668), and GFP (Santa Cruz, #sc-8334). Anti-ALK monoclonal antibodies, mAb46, mAb30, and mAb13, generously provided from Dr. Marc Vigny (INSERM U440, Paris) [31].

#### Drosophila

The gl-tau2.1 line expressing wild-type human tau4R in pExpress-gl modification of the GMR expression vector and showing a moderate phenotype on the third chromosome was obtained from Dr. Daniel Geschwind (University of California-Los Angeles, CA) [32]. UAS-tau fly line for the generation of elav-tau was obtained from Dr. Mel Feany [33]. The wild-type full-length *Drosophila* Alk fly line, UAS-Alk^FL^ and *Drosophila* Alk mutants fly lines, UAS-Alk^ACT^, and UAS-Alk^DN^ were generously provided from Dr. Ruth Palmer (The Salk Institute, CA) and Dr. Manfred Frasch (Mount Sinai School of Medicine, NY), respectively [34–36]. The constitutively active ALK encodes a fusion construct of codons 1–117 of human nucleophosmin and codons 1129–1701 of *Drosophila* Alk. The dominant-negative (DN) ALK construct encodes the extracellular domain, transmembrane domain, and a short tail of the intracellular domain of *Drosophila* Alk. Flies were grown on standard cornmeal-based fly media at 25 °C. Adult flies were used for analysis 5 days post eclosion.

### Mice

WT (C57BL/6), TauC3 (BALB/c), 3xTg-AD (C57BL/6), and *Alk* knockout mice (C57BL/6N) were used. TauC3 mice that express a caspase-cleaved form of human 0N4R tau (1–420) under the control of the neuron-specific *BAI1-AP4* promoter were produced as previously described [37]. The *Alk* knockout mice were gifts from Ruth Palmer (University of Gothenburg, Gothenburg, Sweden). Mice were raised under a 12:12 h light–dark cycle with free access to food and water ad libitum. The age-matched same sex was randomly assigned to experimental groups in most in vivo tests. *Alk* knockout mice and their wild-type littermates were used. Both female and male mice were used for experiments. The ages of mice used in each experiment were denoted in the legend. All experiments involving animals were performed according to the protocols approved by the Seoul National University Institutional Animal Care and Use Committee (SNU IACUC) guidelines.

### Lentivirus production

Lentiviral vector stock was produced in HEK293FT cells following calcium phosphate-mediated transfection of the modified transfer vector, packing vectors pMDLg/pRRE, and pCMV-VSV-G-RSV-Rev. Supernatants were harvested over 48–60 h and concentrated by ultracentrifugation at 50,000 × *g* for 2 h at 4 °C. Virus titers were assessed by transducing HEK 293T cells with serial dilutions of viral stock.

### Stereotaxic injection

Lentivirus expressing CSII-EF-MCS-IRES2-Venus-ALK.Fc and control virus were used for the injection. The lentivirus (2.27 × 10^9^ TU/ml, TU; transduction unit) was stereotaxically injected bilaterally into the dentate gyrus (2 μl per hemisphere at 0.4 μl/min). The following coordinates were used to target the DG: anteroposterior = 2.1 mm from bregma, mediolateral = ±1.8 mm, dorsoventral = 2.0 mm. All experiments involving animals were performed according to the protocols approved by the SNU IACUC guidelines.

### Behavior tests

The behavioral tests were blind during the performance and results analysis. In the case of injuries such as wounds during the behavioral experiment, the subject was excluded and the experiment was conducted. These criteria of subject exclusion were pre-established.

#### Y maze spontaneous alternation test

Mice were placed at the end of one arm and allowed to explore freely through the maze (32.5 cm length × 15 cm height) for 7 min. An entry occurs when all four paws are placed into the arm. The number of arm entries and the number of total alterations were recorded and calculated for the percentage. After each trial, the apparatus was cleaned with 70% ethanol [37].

#### 2-Novel object recognition

During the habituation, mice were allowed to move within an empty white plastic chamber (22 cm wide × 27 cm long × 30 cm high) for 7 min at 24 h intervals. After 2 days, mice were exposed to 2 objects and allowed to explore freely for 7 min. In the testing trial performed 24 h later, 1 of the familiar objects was replaced with a novel object and the time spent exploring each object was recorded during a 7 min period. Between each trial, the arena and objects were wiped down with 70% ethanol [37].

#### Passive avoidance task

Passive avoidance chamber is divided into a light compartment and a dark compartment (20 × 20 × 20 cm each) separated by a guillotine door. During the habituation, mice were allowed to freely explore both compartments for 5 min. On the following day, electric foot shock (0.25 mA, 2 s) was delivered to the mice when both hindlimbs of the mice were entered into the dark box. For the test, mice were placed back in the light box 24 h after the conditioning. The latency time for the mice to enter the dark compartment was measured with a 7-min cut-off point [37].

### Statistical analysis

Statistical analyses were conducted using Prism. No statistical methods were used to predetermine sample sizes, but the sample sizes used are similar to those generally employed in previous publications. Data distributions were tested for normality using D’Agostino-Pearson test. The homoscedasticity of data was tested by *F*-test or Bartlett’s test. All results are presented as mean ± SEM or mean ± SD Statistical comparisons between two groups were performed using two-tailed Student’s *t* test or Bonferroni *t*-test and comparisons between multiple groups were performed using one-way analysis of variance (ANOVA) followed by Tukey’s test as appropriate. Significance is reported as *P < 0.05, **P < 0.01, ***P < 0.001 and ****P < 0.0001. Not significant values are not denoted except for emphasis. The experiments were blind and the data were collected and processed randomly.

## Results

### Neuronal ALK promotes the accumulation and aggregation of phosphorylated tau

To identify novel regulators functioning in tau-mediated pathogenesis, we established a cell-based tau aggregation assay. Ectopic expression of GFP-tagged and caspase-cleaved tau (GFP-tau_D421_) resulted in the formation of green fluorescence- and thioflavin S-positive tau aggregates in HT22 mouse hippocampal neuronal cells (Fig. S1a). Utilizing this assay, we functionally screened ~2600 cDNAs encoding 630 kinases and 2000 membrane proteins, and isolated a list of putative positive clones, including ALK, AK1 [38], and IRE1 [39], that affected tau aggregation following ectopic expression of these cDNAs (Fig. S1b). Among them, mouse ALK, a receptor on the plasma membrane, effectively increased tau aggregation in the transfected cells (Fig. S1c).

**Fig. 1:**
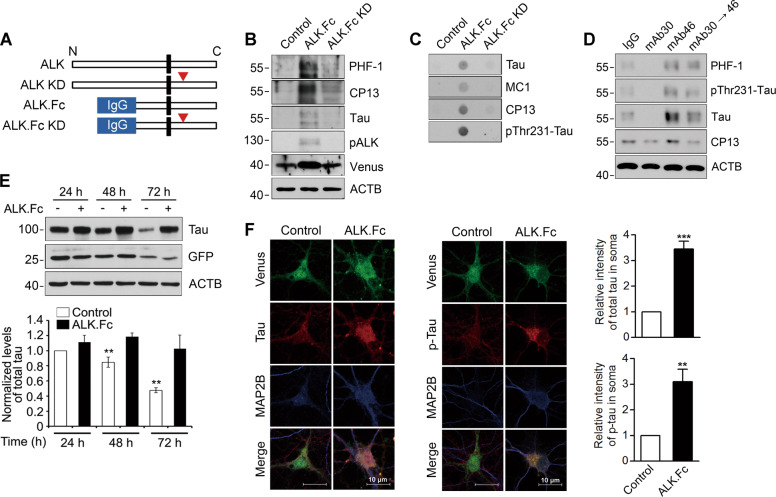
ALK enhances tau accumulation in primary neurons. **A** Schematic diagram of the wild-type and mutant ALK constructs. Arrowheads indicate kinase-dead (KD) mutants, and IgG Fc is the Fc region of immunoglobulin G. **B–E** ALK-induced increases in tau accumulation, phosphorylation, and aggregation. Mouse primary hippocampal neurons (DIV 13) were infected with control vector, ALK.Fc or ALK.Fc KD lentivirus (MOI5) and maintained for 8 days in vitro (**B**, **C**). Mouse primary hippocampal neurons (DIV 7) were treated with 6 nM IgG, mAb30 (antagonistic antibody), mAb46 (agonistic antibody) or mAb46 following pre-incubation with mAb30 for 24 h (**D**). HT22 cells were transfected with GFP-tau and control vector (−) or ALK.Fc (+). The relative ratios of the tau to β-actin signal were quantified by densitometric analysis (*n* = 3). Bars depict mean ± SD. **P < 0.01; paired two-tailed Student’s *t*-test (**E**). **F** ALK activation leads to tau relocalization in cultured neurons. Mouse primary hippocampal neurons (DIV 7) were infected for 2 days with control lentivirus or ALK.Fc lentivirus, and immunostained with anti-MAP2B (blue) or anti-tau (red) antibody, after which fluorescent signals were observed under a confocal microscope (**F** left and middle). The relative immunoreactivity of total tau (Tau) and phosphorylated tau (p-Tau) was quantified by densitometric analysis (*n* = 10) (**F** right). Bars depict mean ± SEM. Tau, *P* = 0.0009; p-Tau, *P* = 0.0025; paired two-tailed Student’s *t* test.

Like mouse ALK, infection of primary hippocampal neurons with a lentivirus carrying constitutively active ALK.Fc, a chimeric protein in which the extracellular domain of human ALK was replaced by the mouse IgG 2b Fc domain for dimerization [40] (Fig. 1A), dramatically increased accumulation of phosphorylated tau (Fig. 1B, C). By contrast, the kinase-dead ALK.Fc KD mutant exhibited no such stimulatory activity. Likewise, treating primary hippocampal neurons with mAb46, an agonistic ALK antibody [40], increased tau levels and its phosphorylation, while pre-treatment with mAb30, an antagonistic ALK antibody [40], blocked mAb46-mediated tau accumulation (Fig. 1D). We confirmed that both mAb46 and mAb30 antibodies well detected mouse ALK in the transfected cells (Fig. S1d). That overexpressed ALK did not affect tau mRNA levels (Fig. S1e), suggests that the increase in tau protein levels (approximately fivefold) by ALK reflects a posttranslational event. This was confirmed by our observation that ALK.Fc enhanced the stability of tau protein in HT22 cells (Figs. 1E and S1f).

In AD, tau accumulation is accompanied by abnormal phosphorylation at many epitopes [41]. We found that ALK phosphorylated human and mouse tau at multiple serine and threonine residues, including the AD epitopes PHF-1 (S396/S404), 12E8 (S262/S356), CP13 (S202) and pThr231‐tau (Figs. 1B–D and S1g). In addition, ALK altered the subcellular localization of tau in primary neurons. The phosphorylated tau was apparently relocalized from the axon to the somatodendritic region following the expression of ALK.Fc (Fig. 1F). Besides, genetic ablation of *Alk* might reduce phosphorylated tau and total tau protein levels in the mouse cortex and hippocampus (Fig. S1h). Collectively, these findings indicate that ALK stimulates tau stabilization and hyperphosphorylation in neuronal cells through its kinase activity.

### ALK impairs autophagosome maturation to accumulate tau protein via Grb2

**Fig. 2:**
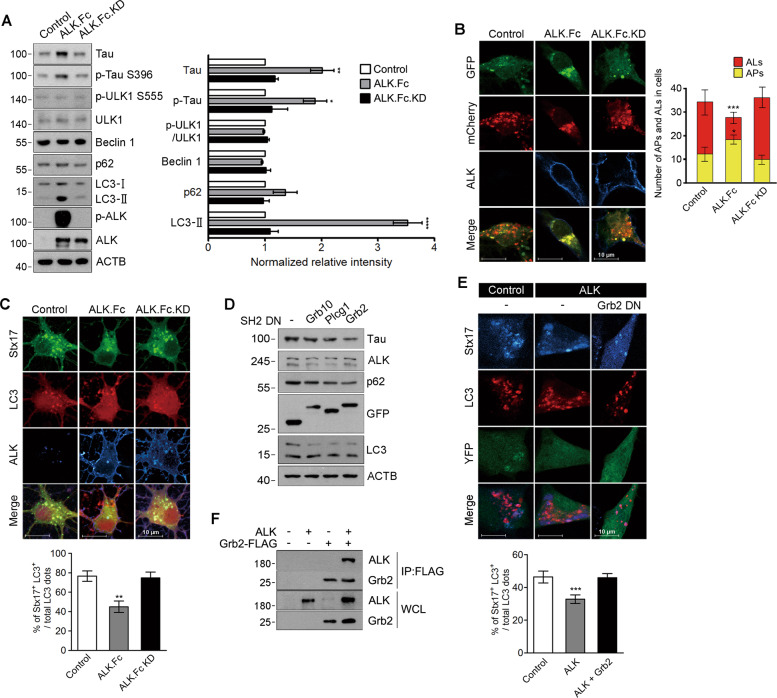
ALK impairs autophagosome maturation in a Grb2-dependent manner. **A** ALK causes LC3-II accumulation without affecting ULK1/Beclin 1 activity. SH-SY5Y cells expressing GFP-tau were transfected for 24 h with control vector, ALK.Fc or ALK.Fc.KD, and analyzed by western blotting (left). The signals on the blots were quantified by densitometric analysis (*n* = 3) (right). Bars depict mean ± SEM. Tau, *P* = 0.0026; p-Tau, *P* = 0.0399; LC3-II, *P* < 0.0001; one-way ANOVA followed by Tuckey’s test. **B** ALK impairs autophagic flux. SH-SY5Y cells were transfected for 24 h with mCherry-GFP-LC3 plus control vector, ALK.Fc or ALK.Fc.KD, and observed under a confocal microscope (left). The numbers of GFP (APs)- and mCherry (ALs)-positive dots per cell were quantified (*n* = 30–40 cells) (right). Bars depict mean ± SEM. APs; *P* = 0.0243, ALs; *P* = 0.0002; one-way ANOVA followed by Tuckey’s test. APs autophagosomes, ALs autolysosomes. **C** ALK decreases colocalization of GFP-Stx17 with RFP-LC3. Mouse cortical neurons (DIV 8) were transfected for 24 h with GFP-Stx17, RFP-LC3 and control vector, ALK.Fc or ALK.Fc.KD for (upper). The percentages of GFP-positive RFP-LC3 dots among total LC3 dots (matured autophagosomes) were determined (*n* = 30–50) (lower). Bars depict mean ± SEM. *P* = 0.0013; one-way ANOVA followed by Tukey’s test. **D**, **E** A dominant-negative form of Grb2 (SH2-DN) attenuates ALK-induced tau accumulation and autophagosomal dysfunction. SH-SY5Y cells stably expressing ALK and GFP-tau were transfected for 24 h with control vector (−), Grb10, Plcg1, or Grb2 SH2-DN (**D**). SH-SY5Y/control and SH-SY5Y/ALK stable cells were transfected with BFP-Stx17, RFP-LC3, and control vector (−) or Grb2 SH2-DN (e, upper). The percentages of GFP-positive RFP-LC3 dots among total LC3 dots (matured autophagosomes) were determined (*n* = 30–50). Bars depict mean ± SEM. *P* = 0.0008; one-way ANOVA followed by Tukey’s test (e, lower). **F** Grb2 interacts with ALK. SH-SY5Y cells were transfected for 24 h with Grb2-FLAG, ALK or both Grb2-FLAG and ALK for 48 h, Grb2-FLAG was immunoprecipitated (IP) with FLAG-M2 beads, and the immune complexes were assessed by western blot with an anti-ALK antibody.

The finding that ALK enhances levels of both tau protein and its aggregation led us to ask whether ALK plays a role in the degradation and/or clearance of tau. ALK.Fc expression did not directly affect proteasome and lysosome activities (Fig. S2a–d). Interestingly, ALK.Fc significantly increased levels of p62, an autophagosome substrate, and LC3-I/II, two autophagosome markers [42], as well as tau. Although ALK.Fc did not phosphorylate activatory residue at Ser555 of ULK1, an initiator Ser/Thr kinase of autophagy [43], or did not affect the level and complex formation of Beclin 1, a component of VPS34 complexes [44] (Figs. 2A and S3a), it is likely that ALK impairs autophagic flux by blocking a late step in the autophagic process. As expected, in mCherry-GFP-LC3 assays in which LC3 fused with both acid-insensitive mCherry and acid-sensitive GFP emits a yellow fluorescence (mCherry + GFP) in neutral autophagosomes but emits a red fluorescence (mCherry) in acidic autolysosomes [45], we found that ALK increased numbers of yellow-colored autophagosomes but decreased those of RFP only dots in autolysosomes (Fig. 2B). In addition, ALK.Fc, but not inactive ALK, interfered with the colocalization of GFP-Stx17, a SNARE that guides autophagosomes to lysosomes for fusion [46], with RFP-LC3-positive dots (Fig. 2C). Thus, ALK activation leads to impairment in autophagosome maturation.

Up to now, no downstream mediator of ALK has provided a clue to the mechanism underlying ALK-induced autophagosomal defects. Upon activation, ALK is autophosphorylated at key tyrosine residues in its cytosolic domain to create specific sites for the assembly of downstream signaling adapters harboring Src homology 2 (SH2) and phosphotyrosine binding (PTB) domains [47]. Therefore, we screened 108 DN forms of the proteins to isolate the SH2 domain-containing adapters that affect tau accumulation and identified DN forms of Grb10, Plcg1, and Grb2 (Fig. S2e). Overexpression of those three forms apparently reversed ALK-mediated accumulation of tau and p62, and LC3 conversion (Fig. 2D). Among them, Grb2-DN also restored the colocalization of BFP-Stx17 and RFP-LC3 in SH-SY5Y/ALK stable cells (Fig. 2E). We also found that Grb2 was able to interact with ALK (Fig. 2F). These results demonstrate that ALK interferes with autophagosome maturation in a Grb2-dependent manner, causing tau protein to accumulate in neurons.

### ALK induces deterioration of axons and dendritic spines, making neurons vulnerable to death

**Fig. 3:**
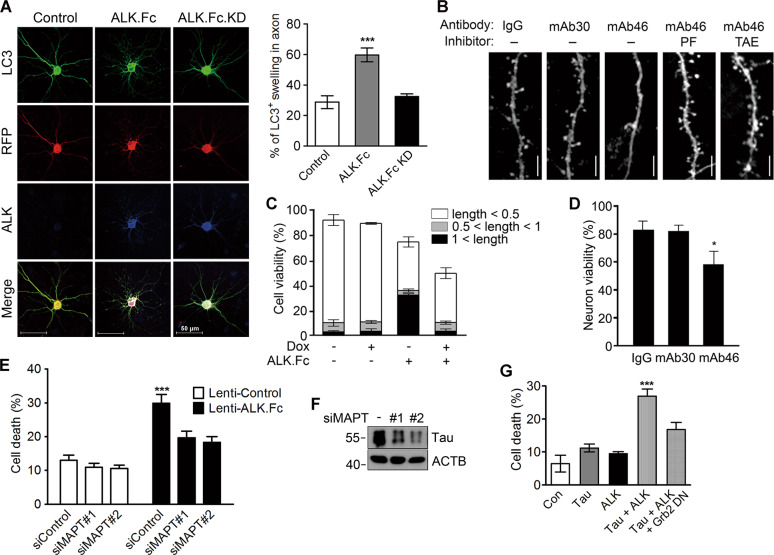
ALK causes axonal swelling, spine loss, and neuronal cell death. **A** ALK induces axonal swelling in primary neurons. Primary cortical neurons (DIV 8) were transfected for 24 h with RFP-LC3 plus control vector, ALK.Fc or ALK.Fc.KD, and observed under a confocal microscope (left). The numbers of neurons with LC3-positive swollen axons were counted (*n* = 60–70). Bars depict mean ± SEM. *P* = 0.0004; one-way ANOVA followed by Tukey’s test (right). **B** An agonistic ALK antibody reduces spine density on primary neurons. Mouse hippocampal neurons (DIV 13) were transfected with GFP for 2 days and then incubated for an additional 12 h with control immunoglobulin G (IgG) or an agonistic (mAb46) or antagonistic (mAb30) monoclonal ALK antibody in the absence or presence of the ALK inhibitors PF-02341066 (PF, 100 nM) and NVP-TAE684 (TAE, 100 nM). Spine dendrites on the neurons were examined under a fluorescence microscope (Scale bar, 10 μm). **C** Tet-inducible tau expression in HT22 cells exacerbates ALK-mediated neuronal cell death and impairs neurite outgrowth. HT22/tet-tau cells were cotransfected for 24 h with GFP-N1 plus control vector (−) or ALK.Fc and then grown for 24 h in the presence (+) or absence (−) of 1 μg/ml doxycycline (Dox). Neurite outgrowth and cell viability were analyzed under a fluorescence microscope. The relative ratios of neurite outgrowth were determined by counting neuronal cells harboring the indicated length of neurites. **D** Agonistic ALK antibody increases cell death among primary neurons. Mouse hippocampal neurons (DIV 13) were transfected with GFP for 2 days and then incubated with immunoglobulin G (IgG), mAb46, or mAb30 antibody for 12 h. Cell viability was determined using ethidium homodimer (*n* = 4–5). *P* = 0.0119; Bonferroni *t*-test. Bars depict mean ± SD. **E**, **F** Tau knockdown attenuates ALK-induced neuronal cell death. Mouse primary cortical neurons (DIV 15) were transfected with 100 nM Control (siControl) or MAPT siRNA (siMAPT) for 12 h and then infected with control lentivirus or ALK.Fc lentivirus (MOI 3) for 48 h. Neuronal cell death was quantified after staining with propidium iodide (*n* = 3, 5 fields, the number of cells per field = 60–100) (**E**) and tau knockdown was confirmed by western blotting using TG5 antibody (**F**). Bars depict mean ± SEM. *P* = 0.0003; one-way ANOVA followed by Tukey’s test. **G** A dominant-negative form of Grb2 (SH2-DN) attenuates ALK-induced cell death. HT22 cells were cotransfected for 48 h with GFP-tau, ALK, and dominant-negative form of Grb2 for 48 h, as indicated. The apoptosis of GFP-positive cells was analyzed by propidium iodide staining under a fluorescence microscope. Bars depict mean ± SEM. *P* = 0.0006; one-way ANOVA followed by Tukey’s test.

When immature autophagosomes fail to fuse with lysosomes, both the autophagosomes and lysosomes pile up [48]. The excess autophagosomes/lysosomes cause axonal swelling, followed by retraction and neuronal death [49]. ALK.Fc caused LC3-positive neurons to undergo massive axonal swelling (Fig. 3A). In addition, the spine density on primary hippocampal neurons was significantly reduced by treatment with the agonistic ALK antibody mAb46, but not with the antagonistic ALK antibody mAb30 (Fig. 3B). The mAb46-induced reduction in spine density was effectively blocked by the ALK inhibitors NVP-TAE684 and PF-2341066 developed by Novartis and Pfizer Inc. [23, 24], respectively (Fig. 3B).

Because neuronal cells undergoing neurodegeneration are susceptible to cell death, we directly assessed the effects of ALK on neuronal cell death. As previously shown in flies [50, 51], we confirmed that neurite outgrowth was promoted by ALK.Fc expression in HT22 cells (Fig. 3C). Intriguingly, this neurite outgrowth induced by ALK was repressed by Tet-inducible tau expression in HT22 cells, and numerous dying cells were observed (Fig. 3C). On the other hand, cell death was not frequently observed among HT22 cells and non-neuronal cells expressing either ALK.Fc or tau alone (Figs. 3C and S3b). Compared to HT22 cells, treatment with the agonistic ALK antibody mAb46-induced a significant amount of cell death among primary hippocampal neurons (Fig. 3D). More, tau knockdown in primary cortical neurons suppressed ALK-induced neuronal cell death (Fig. 3E, F), demonstrating that neuronal toxicity of ALK is dependent on tau. In addition, a DN form of Grb2 attenuated ALK-mediated cell death (Fig. 3G). We, therefore, concluded that by inducing axonal deterioration and reductions in spine density, ALK reduces neuronal viability in a tau-dependent manner.

### Neuronal ALK exacerbates the rough eye phenotype and shortens lifespan in tau fly models

**Fig. 4:**
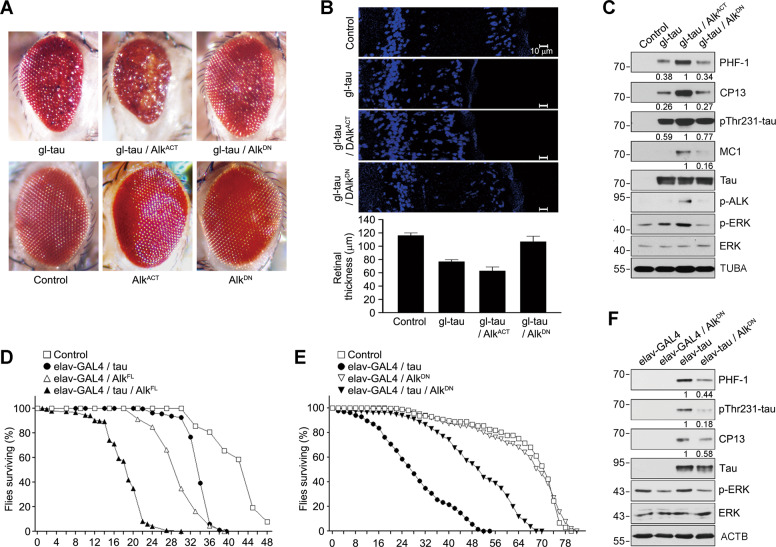
ALK regulates tau-mediated neurodegeneration in tauopathy fly models. **A** Enhancement of the rough eye phenotype and disordered ommatidial morphology in tau transgenic flies expressing active *Drosophila* Alk (Alk^ACT^). External phenotypes of fly eyes were examined under a microscope. Control and experimental genotypes: Control (GMR-GAL4/+), Alk^ACT^ (GMR-GAL4/+; UAS-*Alk*^ACT^/+), Alk^DN^ (GMR-GAL4/+; UAS-*Alk*^DN^/+), tau (GMR-GAL4/+; gl-tau2.1/+), tau + Alk^ACT^ (GMR-GAL4/+; gl-tau2.1/+; UAS-*Alk*^ACT^/+) and tau + Alk^DN^ (GMR-GAL4/+; gl-tau2.1/+; UAS-*Alk*^DN^/+). **B** Exacerbation of retinal degeneration by ALK in tau flies. Internal retinal sections were stained with Hoechst 33342, after which they were observed under a fluorescence microscope (upper). Retinal thickness was quantified (*n* = 3) (lower). **C** Increased tau phosphorylation in tau- and Alk^ACT^-expressing flies. Heads of the flies expressing tau alone or together with an Alk mutant were examined by western blotting. The signals of total tau and phosphorylated tau on the blots were quantified by densitometric analysis and the values are noted under the blot. **D** Shortened lifespan of transgenic flies expressing tau and Alk^FL^ in neurons. At least 200 flies of each genotype were collected and assayed for longevity. Control and experimental genotypes: Control (elav-GAL4/+), tau (elav-GAL4/+; UAS-tau), Alk^FL^ (elav-GAL4/+; UAS-*Alk*^FL^) and tau + Alk^FL^ (elav-GAL4/+; UAS-tau; UAS-*Alk*^FL^). **E** Extended lifespan of transgenic flies expressing tau and Alk^DN^ in neurons. Control and experimental genotypes: Control (elav-GAL4/+), tau (elav-GAL4/+; UAS-tau), Alk^DN^ (elav-GAL4/+; UAS-*Alk*^DN^) and tau + Alk^DN^ (elav-GAL4/+; UAS-tau; UAS-*Alk*^DN^). **F** Reduction of phosphorylated tau by Alk^DN^ in tau flies. Heads of transgenic flies were examined by western blotting (30 days post eclosion). The signals of total tau and phosphorylated tau on the blots were quantified as described in (**C**).

To assess in vivo effects of ALK on tau-mediated neurodegeneration, we employed a human tau transgenic fly (gl-tau2.1 line), a well-characterized tauopathy model in which human tau is expressed in the photoreceptor neurons [32]. As reported, the gl-tau2.1 fly exhibited neurodegeneration and mild disorganization of the retina with disordered ommatidia and bristle abnormalities (Fig. 4A). In the state that *Drosophila* Alk is expressed in the adult CNS, *Alk* transgene was then expressed using the binary GAL4/UAS system in cell populations overlapping those expressing tau [52, 53]. Compared to gl-tau2.1 flies, the eyes of transgenic tau/Alk^ACT^ flies expressing constitutively active *Drosophila* Alk (Alk^ACT^) were much reduced in size and displayed a stronger rough phenotype (Fig. 4A). The rough eye phenotype was significantly alleviated by the expression of DN *Drosophila* Alk (Alk^DN^) in tau/Alk^DN^ flies. Correspondingly, the internal retinal architecture was disrupted in tau/Alk^ACT^ flies, exhibiting severely reduced thickness and marked neuronal loss (Fig. 4B). The retinal architecture was rescued in tau/Alk^DN^ flies and appeared similar to that in control flies. Compared to the flies expressing tau alone (gl-tau), tau phosphorylation (PHF-1, CP13, pThr231, and MC-1) was greatly elevated in the heads of flies expressing both Alk^ACT^ and tau (Fig. 4C). In addition, phosphorylation/activation of both ALK and ERK1/2 was observed in the flies expressing Alk^ACT^, but not in those expressing Alk^DN^. These results demonstrate that ALK activity is critical for tau hyperphosphorylation and contributes to the neurodegeneration seen in tau transgenic flies.

The ALK-induced loss of the photoreceptor neuron phenotype in tau transgenic flies could be caused by progressive neurodegeneration or by developmental perturbation of cell patterning, which often leads to secondary neuronal cell death. To exclude the possibility of secondary effects caused by developmental defects, we used an elav-GAL4 driver to express human tau specifically in postmitotic neurons and then measured the lifespan of the flies. While the lifespan of elav-GAL4/UAS-tau flies and elav-GAL4/UAS-Alk^FL^ flies was moderately shorter than that of control flies, the lifespan of tau/Alk^FL^ flies was largely shortened (Fig. 4D). Conversely, Alk^DN^ expression dramatically extended the lifespan of flies expressing tau, though Alk^DN^ alone had no effect on the lifespan of the flies (Fig. 4E). Moreover, tau phosphorylation (PHF-1, pThr231, and CP13) detected in elav-GAL4/UAS-tau flies was also suppressed by coexpression of Alk^DN^ (Fig. 4F). These results suggest that ALK activity is crucial for tau-induced neurodegeneration in vivo.

### Increased ALK exacerbates memory impairment in 3xTg-AD mice

**Fig. 5:**
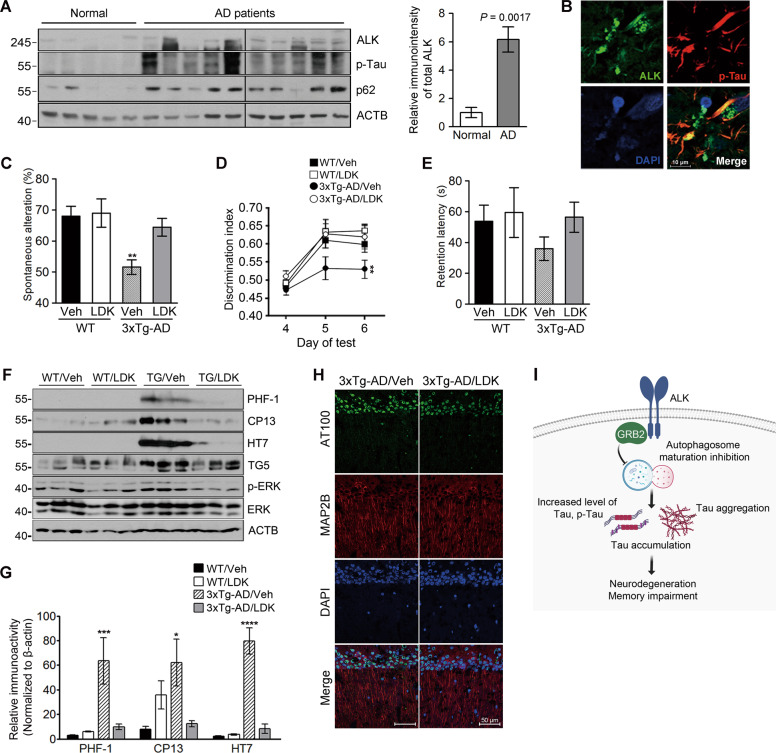
ALK is upregulated in AD brains and pharmacological inhibition of ALK rescues memory impairment in 3xTg-AD mice. **A** Increased expression of ALK and accumulation of p62 in AD brains. Hippocampal lysates from normal elderly controls and patients with AD were analyzed by western blotting (left). Total ALK levels were quantified and normalized to β-actin. Bars depict mean ± SEM; unpaired two-tailed Student’s *t* test (right). **B** Colocalization of ALK and phosphorylated tau within the cortex of patients with AD. **C–E** LDK378 rescues memory in 3xTg-AD mice. Six-month-old 3xTg-AD mice were intraperitoneally injected with 5 mg/kg LDK378 or DMSO (Vehicle) (*n* = 7–9) daily for 4 weeks and then analyzed using Y-maze (**C** *P* = 0.0027), novel object recognition (**D** *P* = 0.0046) and passive avoidance (**E**) tests. **F–H** LDK378 reduces tau phosphorylation and accumulation in the brains of 3xTg-AD mice. Hippocampal extracts from the mice analyzed in (**C**–**E**) were examined by western blotting (**F**). Levels of total tau (TG5), exogenous human tau (HT7), phosphorylated tau (PHF-1, CP13), p-ERK, and ERK on the blots were quantified and normalized to β-actin. Bars depict mean ± SEM. PHF-1, *P* = 0.0059; CP13, *P* = 0.0319; HT7, *P* < 0.0001; one-way ANOVA followed by Tukey’s test (**G**). The CA1 region in the hippocampus of 8-month-old 3xTg-AD/Vehicle and 3xTg-AD/LDK378 mice were immunostained with anti-MAP2B and AT100 antibodies (Representative confocal images, 200×, Scale bar, 50 μm) (**H**). **I** The proposed model of ALK-mediated tau accumulation.

ALK expression is ubiquitously found in the adult mouse brain, including cortex and hippocampus (Fig. S4a), and the purified hippocampal neurons (Fig. S4b). When we analyzed ALK expression in the brains of patients with AD, we found that ALK was elevated by fivefold in the hippocampal tissues in patients with AD, as compared to elderly controls, and this elevation of ALK showed a correlation with tau phosphorylation and p62 accumulation (Figs. 5A and S4c). In addition, immunohistochemistry of the cortical region of patients with AD revealed that ALK immunoreactivity colocalized with the neurofibrillary tangles detected by an antibody against phosphorylated tau (Fig. 5B). ALK is thus aberrantly increased in the AD brains showing tau and p62 accumulation.

We next addressed the effect of increased ALK on AD pathology using 3xTg-AD model mice which express human APP KM670/671NL (Swedish), MAPT P301L, and PSEN1 M146V in neurons [54, 55]. We designed a lentivirus encoding both Venus and ALK.Fc [51] and stereotaxically injected the ALK.Fc-lentivirus into the dentate gyrus of 5- to 6-month-old 3xTg-AD mice. Behavioral tests performed by the mice one month after the injection revealed that spatial memory was greatly impaired by delivery of the ALK.Fc-lentivirus (Fig. S4d). Similarly, a novel object recognition test and a passive avoidance test revealed exacerbated reductions in object recognition memory and the discrimination index in 3xTg-AD mice (Fig. S4e, f). Unexpectedly, we observed noticeable memory impairment in age-matched wild-type (WT) mice after injection with ALK.Fc-lentivirus (Fig. S4d–f). Using western blot analysis, we found increased tau phosphorylation (CP13) after ALK.Fc expression in the hippocampal tissues of both WT and 3xTg-AD mice (Fig. S4g). These results suggest that the increased ALK level worsens memory function in 3xTg-AD mice and increases abnormal tau phosphorylation.

### Pharmacological inhibition of ALK reverses tau pathologies in two AD model mice

We also tested whether ALK inhibition would affect memory function in two models of AD: TauC3 and 3xTg-AD mice. Transgenic mice expressing TauC3 in neurons exhibit rapidly developing memory impairment at a young age with a concomitant increase in tau oligomers and contributes to progressive supranuclear palsy pathogenesis [37, 56]. Because PF-2341066 does not cross the brain–blood barrier (BBB), we directly delivered the ALK inhibitor into the brains of TauC3 mice through intracerebroventricular injection [57]. After 3 weeks of injections, the results of behavioral tests revealed that PF-2341066 blocked the impairment of spatial memory (Fig. S5a), object recognition memory (Fig. S5b), and the discrimination index otherwise seen in TauC3 mice (Fig. S5c). In addition, western blotting showed that the tau phosphorylation (PHF-1, pThr231, CP13, 12E8) observed in control TauC3 mice was inhibited by the treatment with PF-2341066 (Fig. S5d). Thus, ALK plays a role in the impaired memory function and abnormal tau phosphorylation in TauC3 model mice.

LDK378 is a recently developed ALK inhibitor that is highly permeable to the BBB and used in the treatment of brain cancer [58]. We tested the effect of LDK378 on the memory function in 6-month-old 3xTg-AD model mice. The mice were intraperitoneally injected with LDK378 daily for 4 weeks and then analyzed. Consistent with the results observed in TauC3 mice, LDK378 dramatically rescued the memory functions in 3xTg-AD mice, as assayed using the Y-maze (Fig. 5C), novel object recognition (Fig. 5D), and passive avoidance (Fig. 5E) tests. Amyloid-beta immunoreactivity was not altered in the brains of LDK378-injected 3xTg-AD mice (Fig. S5e). At the same time, the results of western blot and immunohistochemistry analysis proved that LDK378 administration inhibited tau phosphorylation at multiple epitopes (PHF-1, CP13, AT100) as well as tau accumulation (HT7) in the brains of the mice (Fig. 5F–H). All these results point to the critical role played by ALK in the phosphorylation and accumulation of tau and in the associated memory impairment seen in 3xTg-AD mice.

## Discussion

Up to now, genome-wide functional screening for mediators regulating the pathogenesis of tauopathies has achieved only limited success. We, therefore, established a cell-based tau aggregation assay using TauC3-GFP. TauC3 aggregates faster than wild-type tau [28] and is localized within tau aggregates in P301S, rTG4510, and TauC3 mice [59–61]. To identify a regulator that affects tau aggregation, we utilized TauC3-GFP aggregation assays and screened a cDNA library encoding thousands of membrane proteins. We employed gain-of-function screening using cDNA over loss-of-function screening using siRNA or sgRNA because the latter may not effectively identify a regulator if the signal is not operating. Overexpressed ALK was activated through autophosphorylation as expected [14, 35] and affected tau aggregation.

ALK is highly expressed from the embryo stage through postnatal day 7, and a minimum level is maintained into adulthood in the brain of mice [17] and is also detected in embryo and adults of *Drosophila* [34–36, 52]. It is noteworthy that ALK is upregulated in the brains of patients with AD and its sustained activation in postmitotic neurons in flies and cultured differentiated neurons leads to deteriorating changes in those neurons. In general, it is known that ALK mainly functions in neural differentiation during development and aberrant activation of ALK as a consequence of the genetic mutation stimulates cell proliferation, providing a driving force for tumorigenesis in peripheral tissue. In our model, the persistent pressure caused by ALK activation in postmitotic neurons may be a risk factor for pathophysiological processes through tau regulation. This would be analogous to what is observed with histone deacetylase, inhibitors of which show promise as anticancer agents and for the prevention of neuronal loss [62, 63].

*Alk* knockout mice have a full-lifespan and show no obvious tissue abnormalities. Two conflicting studies reported about the role of ALK in hippocampal neurogenesis and behavioral task of *Alk* knockout mice [17, 64]. In addition, it was shown that ALK mediates maturation of the newborn neuron via pleiotrophin secreted by adult neural stem cells [65], and regulates the amplitude of spontaneous excitatory postsynaptic currents and GABA transmission in the affected neurons [66, 67]. Similarly, *Caenorhabditis elegans* Alk was proposed to destabilize presynaptic differentiation, while the loss of *D. melanogaster* Alk fails in normal midgut development [34, 68, 69]. ALK inhibition in adult α/β mushroom body neurons promotes long-term memory formation, whereas its overexpression impairs it [70]. Thus, the functions of ALK in the brain might depend on brain regions, neuron types, maturation stages of neurons as well as the strength of ALK signaling, which needs to be clarified more in the future. We here demonstrated the pathogenic role of ALK in mature primary neurons and 7 to 8-month-old mice.

A few studies have previously reported tau regulators, including mTORC1, Nuak1, and Fyn [41, 71, 72]. Fyn, in particular, is known to promote translation and somatodendritic localization of tau in neurons [41]. We also observed that overexpressed Fyn interacts with ALK and that Fyn inhibitors, PP1 and PP2 targeting several members of the Src-family [73], partially reversed the effect of ALK on tau accumulation. However, we could not assure evidence showing the regulation of Fyn and mTORC1 by ALK in tau accumulation (data not shown).

It is exciting to find out that ALK regulates the autophagy-lysosome pathway in neuronal cells. While tau is known to be degraded through both the ubiquitin/proteasome system and the autophagy-lysosomal system [74], autophagy is the main process of tau degradation in primary neurons [7]. Indeed, autophagic and lysosomal defects have been reported in tauopathies. Patients with familial AD, corticobasal degeneration, or progressive supranuclear palsy display increases in p62 and LC3-positive vacuoles and impediment of retrograde transport, which is indicative of impaired autophagic flux and accumulation of immature autophagosomes [75]. Here, we placed ALK as the most upstream receptor in a signaling cascade leading to autophagosomal defects and tau accumulation. We found Grb2 as a downstream adapter of ALK in this signaling pathway. Grb2 was previously shown to be activated by ALK for cancer cell transformation [76] and to interact with UVRAG, a regulator of the early stages of autophagy and autophagosomal maturation [77, 78]. However, ALK.Fc did not affect the interaction of Beclin 1 with UVRAG. It is still unclear yet which stage of autophagosomal maturation is affected by the ALK-Grb2 axis.

Transcriptome data provided by Human Brain Transcriptome showed that ALK expression increases with aging in the hippocampus and neocortex regions [79]. Accordingly, we found that ALK is upregulated by 2- to 3-fold in HT22 cells exposed to various aging/stress signals, such as hydrogen peroxide, tunicamycin, or TNF-α (data not shown). A recent study revealed that inhibiting Alk signaling in adult neurons extends lifespan in *Drosophila* [80]. Thus, the increase of ALK in the brains of the old-age might cause tau pathology. More, RNAseq analysis also has shown significant upregulation of ALK expression in the temporal cortex region of patients with AD [1]. Another genome-wide association analysis study showed that ALK SNP is one of the top-ranked SNPs significantly associated with sporadic amyotrophic lateral sclerosis (ALS) [81]. Given ALS is largely attributable to the accumulation of the misfolded or aggregated proteins, the ability of ALK to impair autophagy function may also contribute to the pathogenesis of other neurodegenerative diseases as well as tauopathies including AD.

ALK has emerged as an attractive target for small-molecule therapy in cancer. LDK378 was approved as a lung cancer therapeutic in 2014 and a therapeutic against brain cancer [25]. Our present finding that ALK transmits tau-mediated neurodegeneration provides a new opportunity for AD therapeutics. In particular, our findings suggest that an antagonistic ALK antibody or small-molecule antagonist is potentially useful for the treatment of tau pathology in AD.

## Supplementary information

Supplemental Figure 1

Supplemental Figure 2

Supplemental Figure 3

Supplemental Figure 4

Supplemental Figure 5

Supplemental Figure legends
