# Supplementary material for: Aberrant role of ALK in tau proteinopathy through autophagosomal dysregulation
Source: Mol Psychiatry. 2021 Jan 15;26(10):5542–56. doi: 10.1038/s41380-020-01003-y (PMC8758490; doi:10.1038/s41380-020-01003-y)
Supplement: Supplementary file 6 — Supplemental Figure legends [file 41380_2020_1003_MOESM6_ESM.docx]

**Supplemental Figure legends**

**Supplemental Figure 1. ALK increases tau aggregation and phosphorylation in HT22 mouse hippocampal cells and mice.**

**a,** Overexpression of ALK enhances PHF1- and thioflavin S-positive tau aggregation in mouse hippocampal HT22 cells. HT22 cells were cotransfected for 24 h with HA-tau plus control vector or ALK, and then stained with Thioflavin-S or PHF1 antibody. **b,** Result of the secondary tau aggregation screening. HT22 cells were co-transfected for 24 h with tau-GFP and 51 positive clones that were isolated from the primary screening. Then, numbers of cells showing tau aggregates were counted under a fluorescence microscope. A list of the positive genes, including ALK, AK1, GSK3β and pcDNA (Control) are denoted. **c,** Enhancement of tau aggregation by mouse ALK (mALK). HT22 cells were cotransfected for 24 h with GFP-tau and increasing amounts of a GSK3β dominant-active (DA) mutant or mALK, after which the number of GFP-positive cells was counted. Bars depict mean ± S.D. **P* < 0.05 ***P* < 0.01; paired two-tailed Student’s *t*-test. **d,** Specificity of the agonistic and antagonistic ALK antibodies. HT22 cells were transfected for 24 h with control vector or ALK, and immunoblotted with an agonistic (mAb46) and antagonistic (mAb30) monoclonal anti-ALK antibody. **e,** Mouse primary cortical neurons (DIV 15) were infected with Control or ALK.Fc lentivirus for 72 h. Total mRNA was isolated and analyzed for *Mapt* and *Alk* mRNA levels by qRT-PCR. Used tools are Power SYBR green mixture, Quantstudio3 and *TBP* primers as control target. Bars depict mean ± S.E.M. two-tailed Student’s *t*-test. n.s.; not significant (left). Cell lysates were subjected to western blot analysis (right). **f,** HT22 cells were co-transfected with GFP-Tau and either control vector or ALK.Fc for 16 h and then incubated with 15 mg/ml cycloheximide (CHX) for an additional 36 h. Cell extracts were subjected to western blotting. **g,** Tau hyperphosphorylation is induced by overexpressed ALK. HT22 cells were cotransfected for 24 h with GFP-tau and the indicated forms of ALK, and tau phosphorylation and ALK activation were examined by western blotting. **h,** Cortical or hippocampal tissue lysates of 6-, 9- or 12-month-old *Alk* knockout mice and wild-type littermates were subjected to western blot analysis.

**Supplemental Figure 2. ALK does not directly affect proteasome or lysosome activity.**

**a and b,** Overexpressed ALK does not affect the accumulation of the GFP^u^ degron. HT22 cells were cotransfected for 24 h with GFP^u^ plus control vector, ALK.Fc or ALK.Fc KD, and then subjected to western blotting (a) or observed under a fluorescence microscope (b). **c and d,** Overexpression of ALK has no effect on lysosomal activity. Primary hippocampal neurons were cotransfected for 24 h with LAMP1-GFP plus control vector, ALK.Fc or ALK.Fc.KD, and then stained with DQ-Red BSA for 6 h. The intensity of BODIPY TR-X fluorescence colocalized with LAMP1-GFP puncta was measured using a photoshop program. Bars depict mean ± S.E.M. one-way ANOVA followed by Tukey’s test (c). SH‑SY5Y cells were cotransfected for 24 h with GFP-8, lysosome-targeting sequence-harboring GFP (Perez-Sala et al. 2009), and control vector, ALK.Fc or ALK.Fc.KD, and then subjected to western blotting (d). CTSD, Cathepsin D. **e,** Isolation of a dominant-negative SH2 (SH2 DN) mutant interfering with ALK-induced tau accumulation. SH-SY5Y cells were transfected for 48 h with ALK.Fc, GFP-tau and each of 102 SH2 DN constructs and then analyzed by western blotting.

**Supplemental Figure 3. ALK does not affect Beclin 1/UVRAG complex formation and cell death in tau-expressing non-neuronal cells.**

**a,** SH-SY5Y cells were transfected for 24 h with control vector (-) or ALK.Fc, and then lysates were immunoprecipitated (IP) with anti-UVRAG antibody, and the immune complexes were assessed for the presence of Beclin 1 by Western blot (WB) with anti-Beclin 1 antibody. **b,** Absence of ALK-induced cell death in tau-expressing non-neuronal cells. MCF7 cells were transfected for 48 h with GFP-tau and ALK, ALK KD, ALK.Fc or Caspase-8 (a positive control).

**Supplemental Figure 4. ALK is expressed in adult neurons and exogenous ALK exacerbates memory deficits in 3xTg-AD mice.**

**a and b,** Expression of ALK in the mouse brain subregions and neurons. Brain subregions collected from adult (postnatal month 2) mice were analyzed with western blotting following immunoprecipitation (IP) assays. H.C.; heavy chain of immunoglobulin (a). Oligodendrocytes and cortical neurons were separated from the cortex of embryonic mice using optiprep gradients. Expression of mouse ALK was examined by western blotting (b). **c**, Pearson correlation coefficient among p62, PHF-1 and ALK levels shown in the hippocampal lysates of human patients with AD in Figure 5a. **d-f**, Lentiviral delivery of ALK exacerbates memory impairment in 3xTg-AD mice. Lentivirus carrying control or ALK.Fc was stereotaxically injected into the dentate gyrus of 4- to 5-month-old wild-type (WT) and 3xTg-AD mice. After 30 days, Y-maze (d), passive avoidance (e) and novel object recognition (f) tests were performed. Bars depict mean ± S.E.M. two-tailed Student’s *t*-test. **g,** ALK increases abnormal tau phosphorylation in 3xTg-AD mice. Hippocampal tissue extracts from the mice were analyzed by western blotting. Lentiviral delivery of ALK was confirmed using an anti-Venus antibody.

**Supplemental Figure 5. Pharmacological inhibition of ALK attenuates tau phosphorylation and memory impairments in TauC3 mice.**

**a-c,** Intracerebroventricular injection of PF-2341066 ameliorates memory deficits in TauC3 mice. One-month-old TauC3 and age-matched wild-type (WT) mice were administered 9 mg PF-2341066 or DMSO (Vehicle) via intracerebroventricular injection (*n* = 6 ~ 8). After 3-4 weeks, Y-maze (a), novel object recognition (b) and passive avoidance (c) tests were performed. Bars depict mean ± S.E.M. two-tailed Student’s *t*-test. **d,** ALK inhibition reduces levels of phosphorylated tau in TauC3 mice. Hippocampal extracts from the mice were analyzed by western blotting. **e**, The CA1 region in the hippocampus of 8 month-old 3xTg-AD/Vehicle and 3xTg-AD/LDK378 mice were immunostained with MOAB-2 and anti-synaptophysin antibodies (Representative confocal images, 200x, Scale bar, 50 μm).
